# Feikang granules ameliorate pulmonary inflammation in the rat model of chronic obstructive pulmonary disease via TLR2/4-mediated NF-κB pathway

**DOI:** 10.1186/s12906-020-02964-x

**Published:** 2020-06-03

**Authors:** Liuliu Yang, Minyong Wen, Xiaohong Liu, Kai Wang, Yong Wang

**Affiliations:** 1 The First Affiliated Hospital of Guangzhou University of Chinese Medicine, No. 16 Airport Road, Baiyun District, Guangzhou, 510405 Guangdong China; 2 Guangzhou University of Chinese Medicine, Guangzhou, 510006 Guangdong China

**Keywords:** Chinese herbal medicine, Chronic obstructive pulmonary disease, Inflammatory cytokines, Toll-like receptors

## Abstract

**Purpose:**

Several reports have shown that traditional Chinese medicine could be an alternative therapeutic approach for COPD patients, but the mechanism remains unknown. The present study aimed to examine the effects of Feikang granules in a COPD model rat and investigate the possible mechanisms via Toll-like receptor (TLR)/ nuclear factor kappa B (NF-*κ*B) signaling.

**Methods:**

The COPD model rats were treated with Feikang granules, dexamethasone, or normal saline. The pulmonary function; lung tissue histology; levels of inflammatory cytokines; mRNA levels of TNFα, IL-6, TLR4, and TLR2; and protein levels of TLR4, TLR2, p-IκB, IκB and P65 in lung tissues were evaluated.

**Results:**

The present study confirmed that the pro-inflammatory cytokines, TNF-α, IL-1β, IL-6, and IL-17 levels were elevated and the pulmonary function and morphology are altered in COPD model rats. The TLR2 and TLR4 -mediated NF-kB signaling pathway plays a role in the mechanism of action. Feikang granules, a type of Chinese herbal medicine, significantly reduced LPS induced inflammatory cytokines release from lung tissue and alveolar macrophage in a dose-dependent manner. These medical herbs also prevented TLR2/4 and IκB downregulation and reversed the p-IκB and NF-κB p65 upregulation of the lung tissue in the COPD rats. Feikang granules were also found to protect against pulmonary dysfunction and pathological changes in the COPD rats.

**Conclusion:**

The Chinese herbal medicine formula Feikang granules prevent pulmonary inflammation and improve pulmonary function, suggesting that Feikang granules may be an effective treatment for chronic pulmonary diseases, such as COPD.

## Background

Chronic obstructive pulmonary disease (COPD) is a progressive disease characterized by chronic bronchitis, airway obstruction, and emphysema, which is one of the leading causes of morbidity and mortality [1, 2]. Current treatments with bronchodilators, antibiotics combined with corticosteroids, only relieve the symptoms temporarily, but cannot delay or cure the disease [3]. It has been widely accepted that the central mechanism of COPD pathogenesis is chronic inflammation, including inflammatory cytokines release and immune cells responses [4]. Cigarette smoke and the inflammatory microenvironment of the airway are considered to be the main factors inducing pathological changes of alveolar macrophages in COPD [5]. Cigarette smoke may stimulate the airway epithelial cells and release noxious molecules that bind to cell surface Toll-like receptors (TLRs). TLR function forms part of the innate immune system and induces pro-inflammatory cytokine release. For example, TLR-2 and TLR-4 detect bacteria by recognizing components of bacterial cell walls via lipotoeic acid (LTA, Gram-positive) and lipopolysaccharide (LPS, Gram-negative), respectively [6]. TLR4 signaling pathways involve various transcription factors, including Nuclear factor kappa-light-chain-enhancer (NF-κB), Signal Transducers and Activators of Transcription family of transcription factors (STAT1), Interferon regulatory factors (IRF’s), and activator protein 1(AP1), which are all crucial players in the regulation of inflammatory response [7]. Several studies have reported that the TLR4/NF-kB signaling pathway mediates the pulmonary inflammation in COPD model animals or patients [8–10].

Traditional Chinese medicine usually prescribes several kinds of herbs to treat a disease. The composition of prescriptions varies slightly according to the patient’s condition or individual differences. Several reports have already shown that traditional Chinese medicine could be an alternative therapeutic approach for COPD patients [11], such as Buzhong Yiqi Tang, which could improve exercise capacity, lung function and quality of life [12]. We previously reported that Feikang Granules, a type of Chinese oral medicine, could be a clinical prescription for the treatment of stable COPD [13], this is also supported by other studies that show Feikang Granules can significantly improve the symptoms of cough, expectoration, wheezing and shortness of breath in COPD patients at a stable stage [14]. Although these traditional Chinese herbs have been used in China and East Asia for thousands of years, their biological features, especially the biological effects on a pulmonary cell signaling pathway, are still unknown. Feikang Granules are composed of six traditional Chinese medicines: Five-fingered Peach, Prince Ginseng, Poria Cocos, Atractylodes macrocephala, *Perilla frutescens*, and bitter almond. It mainly treats internal injury, cough, asthma, lung distention, and other diseases. However, the reports of its effects or biological mechanism are limited [15, 16]. A few studies of Feikang Granules on animals have a specific protective effect on vascular endothelial injury in hypoxic pulmonary hypertension [17] and significantly reduce the Transforming Growth Factor β_1_ in COPD model Rats [16]. It has been showed that Chinese medicine Radix Stemonae significantly decreases the concentrations of inflammatory mediators TNF-α, IL-8 and LTB4 of COPD model rats [18]. Whether Feikang Granules alleviate the clinical symptoms in COPD patients also involved in the anti-inflammation mechanism remains unclear. Since inflammation is the leading cause of COPD, and the TLR4/NF-kB signaling pathway mediates pulmonary inflammation, we used COPD model rats to explore the anti-inflammatory mechanisms of Feikang Granules.

## Methods

### Chinese herb materials and preparation of Feikang granules

Feikang (healthy lung) Granules are provided by the Pharmaceutical Department of the First Affiliated Hospital of Guangzhou University of Traditional Chinese Medicine. The Feikang granules are composed of six herbs: Five-fingered Peach (Wu Zi Mao Tao, Ficus hirta) 666.7 g, Prince Ginseng (Tai Zi Shen, Radix Pseudostellariae) 444.4 g, Poria Cocos (Fu Ling) 333.3 g, Atractylodes macrocephala (Bai Shu) 266.7 g, bitter almond (Xin Ren, Armeniacae Amarum Semen) 222.2 g and fried *Perilla frutescens* (Zi Su Zi, Perillafrutescens) 222.2 g. The above six traditional Chinese herbs are decocted three times in 10-fold water for 1 h each time, combined with Decoction and filtered. The filtrate is concentrated to a paste with a relative density of 1.25–1.30 g/mL at 70 °C by decompression. The paste is mixed with soluble starch, dried in a vacuum chamber at 70 °C, and then crushed into fine powder. The filtrate is mixed with soluble starch and lactose, and granulated with 90% ethanol, dried to 1000 g granules, which can be separated and packed.

### Chemicals and reagents

Dexamethasone was purchased from Chenxin Pharmaceutical Co., Ltd. (Shandong, China); Lipopolysaccharide (LPS) was purchased from Sigma-Aldrich (St. Louis, MO, USA); ELISA kits of TNF-α, IL-6, IL-1β, and IL-17 were purchased from R&D system Inc. (Minneapolis, MN, USA); TRIzol, reverse transcription kit, and PCR MasterMix were purchased from TaKaRa Company (Kusatsu, Shiga, Japan); the primary antibodies of TNF-α, IL-6, NF-κB, phosphorylation of IκBα (*p*-IκBα), TLR4, TLR2, phosphorylation of p65 (*p*-p65), rabbit anti-Histone H3 antibody and rabbit anti-GAPDH polyclonal antibody were purchased from Cell Signaling Technology Inc. (Danvers, MA 01923, USA).

### Experimental animals

Twelve-week old male Sprague Dawley rats (220–240 g) were purchased from Guangdong Provincial Laboratory Animal Center (Guangzhou, China). Rats were housed in an air-conditioned room (21 °C ± 2 °C) under a 12-h light/dark cycle and allowed free access to water and standard chow ad libitum. The animals were acclimated for 7 days prior to operation. Animals were reared and handled strictly according to the obligations of the Animal Ethics Committee of Guangzhou University of Chinese Medicine and the guidelines for the care and use of laboratory animals from the National Institute of Health. Thirty SD rats were randomly assigned into six groups: regular group, model group, low, medium and high dose groups (10.11, 20.22, 40.44 g.kg-1) and dexamethasone group (1 mg.kg-1), with 4 ~ 6 rats in each group. Because the control group showed very consistent results while the model group has some variability, we used 4 rats for the control group and 6 rats for the model group. All other groups kept the minimum animal numbers according to our animal policy. The dose of Feikang Granule in rats was converted into an equivalent dose for a 60 kg body weight adult (1.617 g/kg). The low, middle and high doses were equivalent to 1, 2 and 4 times of the clinical dose, respectively. Rats were euthanized using carbon dioxide overdose followed by decapitation. This protocol was approved by the Animal Ethics Committee of Guangzhou University of Chinese Medicine. Experiments were performed at the laboratory center of The First Affiliated Hospital of Guangzhou University of Chinese Medicine.

### COPD model preparation

The rat COPD model was established through continuous smoking combined with the LPS intratracheal drip [19, 20]. The rats were placed in the PAB-S200 passive smoking animal poisoning system (Belanbo Science and Technology Inc., Beijing, China). The top of the box has two small holes with diameters of 1.5 cm. Ten Coconut Tree brand cigarettes (containing 1.2 mg of nicotine, 12 mg of tar and 14 mg of carbon monoxide) were ignited and placed in the poisoning box. After closing the poisoning box for 1 h, the rats were taken out. After 15 min of rest, the rats were placed in the poisoning box again for another hour (including cigarettes). Such 2 h smoking treatment was applied once a day for 20 weeks. On the 1st and 14th day, 200 mL LPS solution (1 mg/ mL) was dripped into the trachea of each model animal. Normal group was dripped with the same amount of saline, and no smoking treatment was given on the same day.

From the fifth week onwards, rats in the low, middle, and high dose groups were given Feikang granules through intragastric administration at the corresponding dosage for 16 weeks, rats in the normal group and control group were given the same amount of saline by intragastric administration, and rats in the dexamethasone group were given intraperitoneal injection at the same dosage 30 min before smoking.

### Sample collection and animal monitoring

#### General condition of rats

During the experiment, the breathing (cough, wheezing, shortness of breath, hair loss, and shedding), drinking and eating, activity and response to external stimuli were observed.

#### Pulmonary function in rats

The pulmonary functions of rats were measured using a noninvasive pulmonary functionality test system (BUXCO MA1320 respiratory function test table (Buxco, Wilmington, North Carolina, USA) to measure the minute ventilation (MV), peak of inspiratory flow (PIF), peak of expiratory flow (PEF), ratio of expiratory/inspiratory time (Te/Ti), tidal volume (TV), and 50% tidal volume expiratory flow (EF50).

#### Collection and detection of bronchoalveolar lavage fluid (BALF) in rats

The left lobe was ligated at the root of the left hilum with fine cotton thread. We cut the neck skin upward along the median line of the thoracic incision, bluntly separated the soft tissue, exposed the trachea, cut the trachea through a T-shaped incision at the thyroid position, intubated the trachea with the plastic tube of the scalp indwelling needle, and slowly injected 0.5 ml PBS into the right lung through the intubation with syringe. It could be seen that the right lung gradually expanded, and the color changed from dark red to pink white. After all PBS injections were completed, we gently massaged the lungs and then withdrew the fluid 10 s later. The process was repeated twice. The recovery rate was about 80%. The withdrawn liquid was transferred into a 1.5 ml sterile EP tube, which was bronchoalveolar lavage fluid.

After repeated cleaning, broncho-alveolar lavage fluid (BALF) was aliquot into three parts. One part was fixed in 4% polyformaldehyde solution and stored at room temperature for histopathological examination; the second part was immersed in RNA later solution and stored in a refrigerator at − 80 °C for qPCR detection; the other part was stored in 1.5 mL EP tube and stored in a refrigerator at − 80 °C for Western Blot detection.

### Pathological examinations of lung tissues

The rats were sacrificed on the last day of experiments after lung function assessments. Lung tissue samples were collected and fixed with 4% paraformaldehyde, routinely dehydrated, embedded in paraffin, and sectioned (5 μm). The specifications of the color kit stained the nucleus and cytoplasm respectively, and turned transparent when dehydrated. After air-drying, neutral gum seals were used. The photographs were observed for each slide under a microscope. As a result of staining, the nucleus’ were blue-purple and cytoplasms were varying degrees of pink. Histopathology was viewed using a light microscope (Leica Microsystems, Wetzlar, Germany) equipped with × 400 magnification.

### Macrophages identification and culture

Separation and identification of macrophages from bronchoalveolar lavage fluid: After centrifugation at 1500 rpm for 10 min, the cells were collected and suspended in a RPMI 1640 basic culture medium. The cells were incubated in an incubator at 37 °C and 5% CO_2_. After 2 h, the macrophages were adherent, and the adherent cells were considered to be alveolar macrophages. (2) The isolated alveolar macrophages were identified by their morphology under a microscope and expression of CD68 by flow cytometry. (3) The identified macrophages were then transferred to the 24-well culture plate, 10,000 cells per well, heavily suspended in 2 ml complete culture medium containing 20% fetal bovine serum; 4% FITC-E.coli 500ul per well was added, incubated in 37 °C incubator for 6 h; The levels of inflammatory factors IL-1β, TNF-α, IL-6 and IL-17 were determined by ELISA.

### Elisa

Rat alveolar lavage fluid was centrifuged for 10 min at 1500 RPM. The supernatant was transferred to another EP tube and stored at − 80 °C. The contents of cytokines IL-1β, TNF-α, IL-6 and IL-17 were determined by ELISA in strict accordance following the kit instructions.

### Real-time quantitative PCR

Total RNA was extracted from lung tissues (100 mg) with TRIzol reagent (Invitrogen). Reverse-transcription was performed with 1000 ng total RNA by using a Takara cDNA synthesis kit (BioRad, USA). After RNA concentration was measured by a nucleic acid quantifier, DNA was synthesized by a reverse transcription kit operation. With GAPDH as the internal reference, PCR amplification was performed by the CFX96 Touch fluorescence quantitative PCR analyzer (primer sequence is shown in Table 1). The relative mRNA levels of genes TNF-α, IL-6, TLR4, and TLR2 were determined with qRT- PCR by using QuantiTect SYBR Green Kit (Qiagen) and calculated using the 2-^ΔΔCt^ method.

**Table 1:** Primer sequence for RT- PCR

gene	Forward	Reverse
TLR2	TCTCCAAGGAAGAATCCTCCAA	GAGCTGCCCTTGCAGATACC
TNF-α	TGGCCCAGGCAGTCAGA	GGTTTGCTACAACATGGGCTACA
IL-6	GCTGCAGGCACAGAACCA	ACTCCTTAAAGCTGCGCAGAA
GAPDH	CCATCTACGAGGGCTACGC	CGGCTGTGGTCACGAAGG

### Western blotting

The total proteins of rat lung tissues were extracted with RIPA lysis buffer. Equal amounts of total proteins (30 *μ*g) were separated in 10% SDS-PAGE, transferred onto PVDF membrane. After 2 h enclosed with PBS containing 5% skimmed milk powder, it was incubated overnight at 4 C with primary antibodies against TLR4, p-IκB, IκB, and NF-κB p65, and then washed with TBST for 4 times, 7 min each time; incubated with the corresponding second antibody at room temperature for 2 h, washed with TBST for 4 times again, 7 min each time; developed with the ECL chemiluminescence method. Image J software was used to calculate the gray value statistical analysis of the strips after development, and GAPDH was used as the internal parameter to calculate the gray ratio of the protein strips and GAPDH strips of each sample. Each experiment was repeated three times.

### Statistical analysis

All data was analyzed by using SPSS 19.0 statistical software and expressed as means ± standards deviation (SDs). Differences between groups were determined by a one-way ANOVA with Tukey multiple comparison tests. Differences were considered significant when *P* < 0.05.

## Result

### Effect of Feikang granule on pulmonary function in rats

**Fig. 1:**
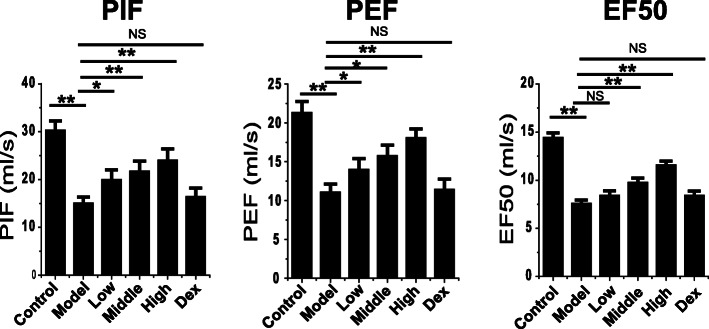
Feikang granules improved pulmonary function in the COPD model rats. The peak of inspiratory flow (PIF), peak of expiratory flow (PEF), and 50% tidal volume expiratory flow (EF50) were measured in all groups of COPD model rats: Normal control rats (con, *n* = 4), COPD model rats without any treatment (Mod, *n* = 6), low (*n* = 5), middle(n = 5) and high (n = 5) dose of Feikang granules groups and dexamethasone group (Dex, n = 5)

The lung function of rats in the normal control group, COPD model group, Feikang granule low dose group, medium dose group, high dose group, and dexamethasone group were examined. The results showed that the indexes of PIF, PEF, and EF50 of lung function in COPD model group decreased significantly (*P* = 0.005, Fig. 1), suggesting that airway resistance increased, airflow was blocked, and lung compliance decreased after a long-term smoking; all doses of Feikang granules could significantly improve the airway hyperresponsiveness of COPD rats (*P* = 0.037) in a dose-dependent manner. The improvement of COPD airway resistance in the dexamethasone group was not significant (*P* = 0.095).

### Feikang granules protect against pathological changes of the lung in COPD rat

**Fig. 2:**
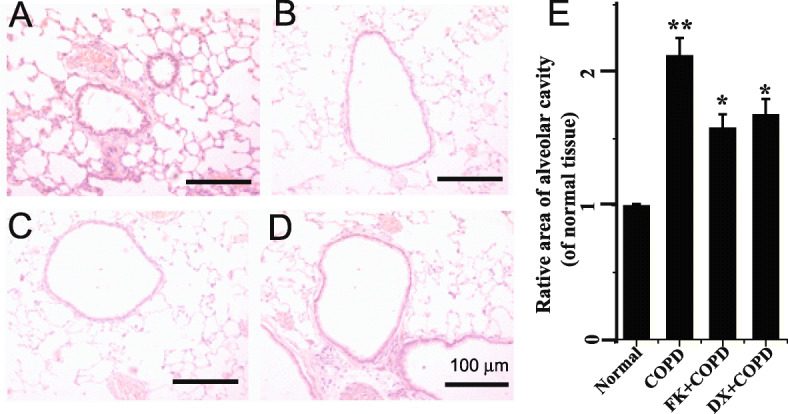
Photographs of HE-stained lung tissues under an optical microscope (× 200). **a** control group; **b** COPD model group; **c** 40.44 g/kg Feikang granules treated group; **d** dexamethasone treated group. **e** Summary results of relative area of alveolar cavity in COPD and Feikang granules or dexamethasone treated rats (of control group)

Histological features revealed the conventional structure of the alveolar cavity in the lung tissues of normal rats. The alveolar wall of the model group became thinner and over-expanded, the number of alveoli decreased, and alveolar fusion and bullae formation were partly seen in the control rats (COPD model rats). Lung tissues manifested histopathological changes, such as edema, inflammatory cell infiltration, and interstitial inflammatory. These pathological changes, especially the alveolar structure, in lung tissues were attenuated by dexamethasone and Feikang granules. The effect of Feikang Granule on pathological changes of lung tissue in COPD model rats is shown in Fig. 2.

### Inhibitory effect of Feikang granule on elevated cytokines levels in the BALF of COPD rat

**Fig. 3:**
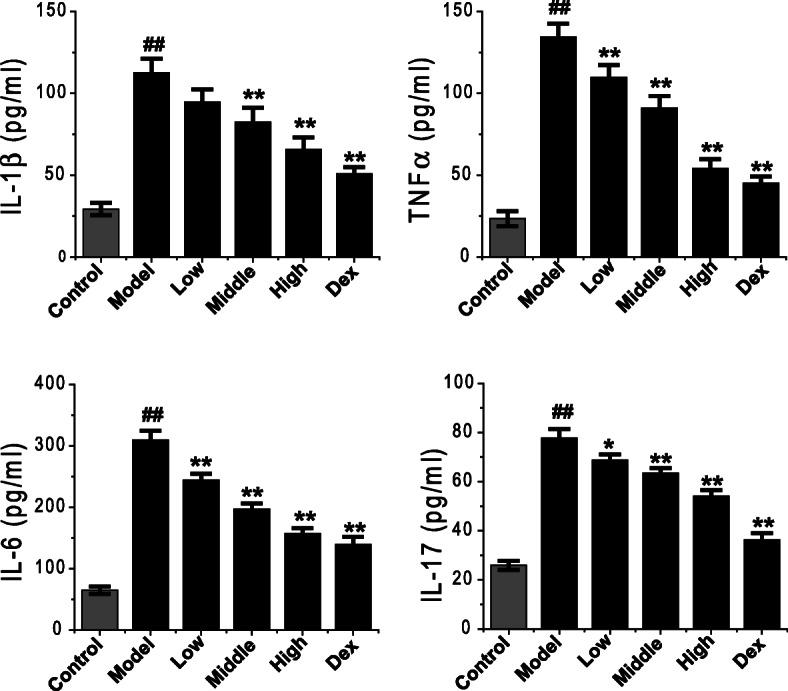
Effects of Feikang granules on inflammatory cytokine changes in bronchoalveolar lavage fluid. Cytokines IL-1β, TNF-*α*, IL-6 and IL-17 were measured. The data are shown as the mean ± the standard deviation (SD). ^*∗*^*p* < 0.05 and ^*∗∗*^*p* < 0.01 indicate statistically significant differences compared to the model group; ^##^p < 0.01 indicate a statistically significant difference in model group compared with normal control group. These results were from the rats in each group of control (n = 4), COPD (n = 6), low dose (n = 5), middle dose (n = 5), high does (n = 5) of Feikang granules and dexamethasone (n = 5)

Consistently with the literature [21, 22], the inflammatory cytokines of IL-1β, TNF-α, IL-6 and IL-17 in the BALF of COPD model rats were significantly increased (Fig. 3 normal control in the blank, COPD control in black, *P* = 0.003). ELISA results showed that the levels of IL-1β, TNF-α, IL-6, IL-17 and other cytokines in BALF of normal rats were very low, while those in BALF of COPD model rats were significantly increased. The levels of IL-1β, TNF-α, IL-6 and IL-17 in BALF of COPD rats decreased gradually with the increase of the dosage of Feikang granules, and the difference was statistically significant (F = 20.43, *P* < 0.001; F = 48.59, P < 0.001; F = 70.57, P < 0.001; F = 66.36, P < 0.001). Moreover, the decrease of IL-1β, TNF-α, and IL-6 in BALF of high dose Chinese herbal medicine group were similar to that of dexamethasone (*P* = 0.123; *P* = 0.299; *P* = 0.210), and the level of IL-17 was significantly different (P < 0.001).

### Effect of Feikang granule on the expression of TNF-α, IL-6, TLR4 and TLR2 mRNAs in lung tissues of COPD rats

**Fig. 4:**
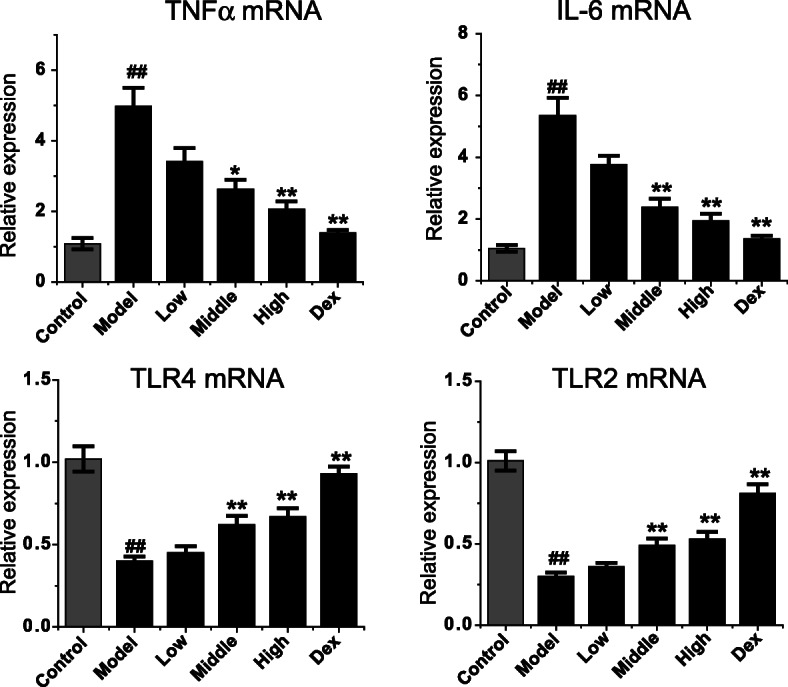
Effects of Feikang granules on inflammatory cytokine mRNA levels in lung tissue. The mRNAs of TNFα, IL-6, TLR4 and TLR2 were measured. The data are shown as the mean ± the standard deviation (SD). ^*∗*^*p* < 0.05 and ^*∗∗*^*p* < 0.01 indicate statistically significant differences compared to the model group; ^##^p < 0.01 indicate a statistically significant difference in model group compared with normal control group. These results were from the rats in each group of control (n = 4), COPD (n = 6), low dose (n = 5), middle dose (n = 5), high does (n = 5) of Feikang granules and dexamethasone (n = 5)

The effect of Feikang Granule on the expression of TNF-alpha, IL-6, TLR4 and TLR2 in lung tissues of COPD model rats is shown in Fig. 3. Compared with the normal group, the expression of TNF-α and IL-6 in lung tissue of COPD model group increased significantly (*P* = 0.003, *P* = 0.005), while the expression of TLR4 and TLR2 decreased significantly (*P* = 0.006, P = 0.005). Compared with the model group, the expression of TNF-α and IL-6 in lung tissue of the middle and high dose groups of Feikang Granules were significantly decreased (TNF-α:*P* = 0.037, *P* = 0.008; IL-6: *P* = 0.009, P = 0.009), while the expression of TLR4 and TLR2 were significantly increased (TLR4: P = 0.006, P = 0.006; TLR2: P = 0.006, P = 0.005). The results showed that Feikang Granule could down-regulate not only the expression of TNF-α and IL-6 in lung tissue of COPD rats but also up-regulate the TLR4 and TLR2 level in a dose-dependent manner (Fig. 4).

### Effect of Feikang granule on cytokines secreted by alveolar macrophages in COPD rats

**Fig. 5:**
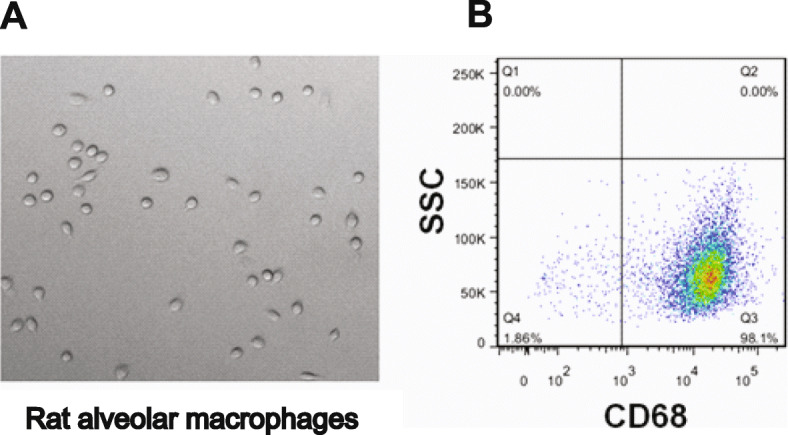
Morphology and flow pattern identification of alveolar macrophages. **a** Represents the microscopic morphology of rat alveolar macrophages (× 400); **b** Represents the flow cytometry identification of rat alveolar macrophages

In order to analyze the effect of drugs on cytokines secreted by alveolar macrophages, alveolar lavage fluids of rats were collected to culture macrophages. The alveolar lavage fluid was cultured in adherent culture, the cells grew round or fusiform adherent to the wall under the microscope, and pseudopodia could extend out. Flow cytometry showed that the adherent cells highly expressed macrophage marker CD68, which was alveolar macrophage.(Fig. 5).

**Fig. 6:**
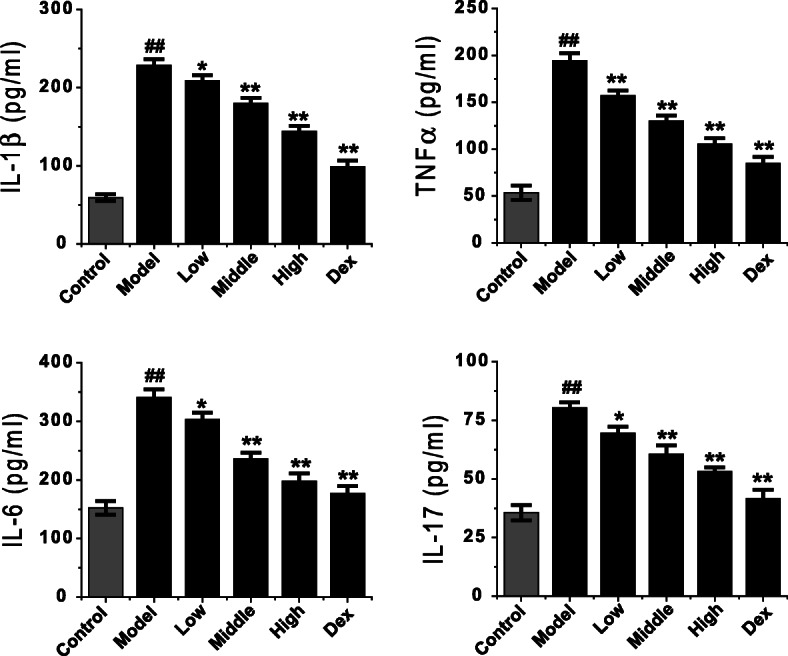
Effects of Feikang granules on inflammatory cytokines secreted by alveolar macrophages in COPD model rats. Cytokines IL-1β, TNF-*α*, IL-6 and IL-17 were measured. The data are shown as the mean ± the standard deviation (SD). ^*∗*^p < 0.05 and ^*∗∗*^p < 0.01 indicate statistically significant differences compared to the model group; ^##^p < 0.01 indicate a statistically significant difference in model group compared with normal control group. All results were repeated from 3 batches of culture mediums

The contents of IL-1β, TNF-α, IL-6, and IL-17 in the supernatant of macrophage culture fluid were measured by ELISA. The results showed that the levels of IL-1β, TNF-α, IL-6 and IL-17 secreted by alveolar macrophages in COPD model rats were significantly higher than those in control group (*P* < 0.001, Fig. 6) like in BALF. Similarly, the inflammatory cytokines secreted by alveolar macrophages in COPD rats treated with Feikang Granule were significantly lower than those in the untreated group. The effect of regulated cytokines in the high-dose group was the most robust, followed by the middle-dose group and low-dose group. The down-regulation of TNF-α, IL-6, and IL-17 secreted by alveolar macrophages in COPD rats was similar to that in dexamethasone group (*P* = 0.324; *P* = 0.208; *P* = 0.957), but the comparison of IL-1β had a statistical difference (*P* < 0.001) (Fig. 6).

### Effect of Feikang granule on the expression of TNF-α, IL-6, TLR4 and TLR2 mRNAs in alveolar macrophages of COPD rats

**Fig. 7:**
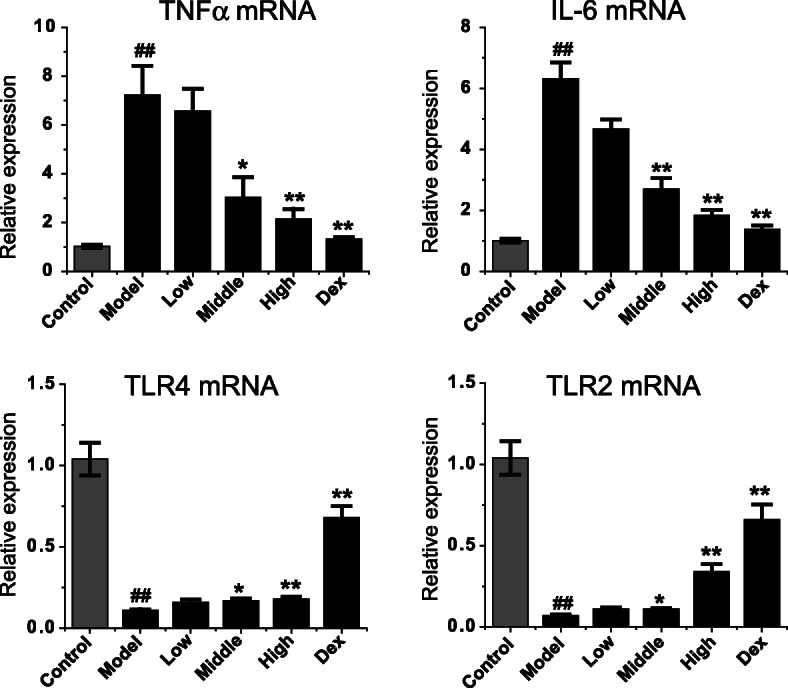
Effects of Feikang granules on inflammatory cytokine mRNA levels in alveolar macrophages of COPD model rats. The mRNAs of TNFα, IL-6, TLR4 and TLR2 were measured. The data are shown as the mean ± the standard deviation (SD). ^*∗*^p < 0.05 and ^*∗∗*^p < 0.01 indicate statistically significant differences compared to the model group; ^##^p < 0.01 indicate a statistically significant difference in model group compared with normal control group. All results were from 3 batches of culture mediums

We extracted the alveolar macrophage RNA from each group and amplified the target genes through reverse transcription. The differences of TNF-α, IL-6, TLR4, TLR2, and mRNA levels in alveolar macrophage of each group were compared. Similarly to the mRNA levels in the lung tissue, TNF-α and IL-6 levels increased, while TLR4 and TLR2 levels decreased in the COPD model rats (Fig. 7). The Chinese herbal medicine Feikang granules showed the down-regulating of TNF-α and IL-6 mRNA levels in alveolar macrophages of COPD rats and up-regulation of TLR4 and TLR2 mRNA levels in a dose-dependent manner. In addition, dexamethasone also significantly decreased the expression of TNF-α and IL-6 in COPD rat pulmonary macrophages, which was similar to the high-dose Feikang granule group (*P* = 0.502; *P* = 0.396), and significantly increased the expression of TLR4 and TLR2. The expression level of TLR2 was similar to that in the high-dose group (*P* = 0.091), while the expression level of TLR4 was higher than that in the high-dose group (*P* = 0.001).

### Effect of Feikang granules on the expression of TLR4, TLR2, p-IκB, IκB and P65 in lung tissues of COPD rats

**Fig. 8:**
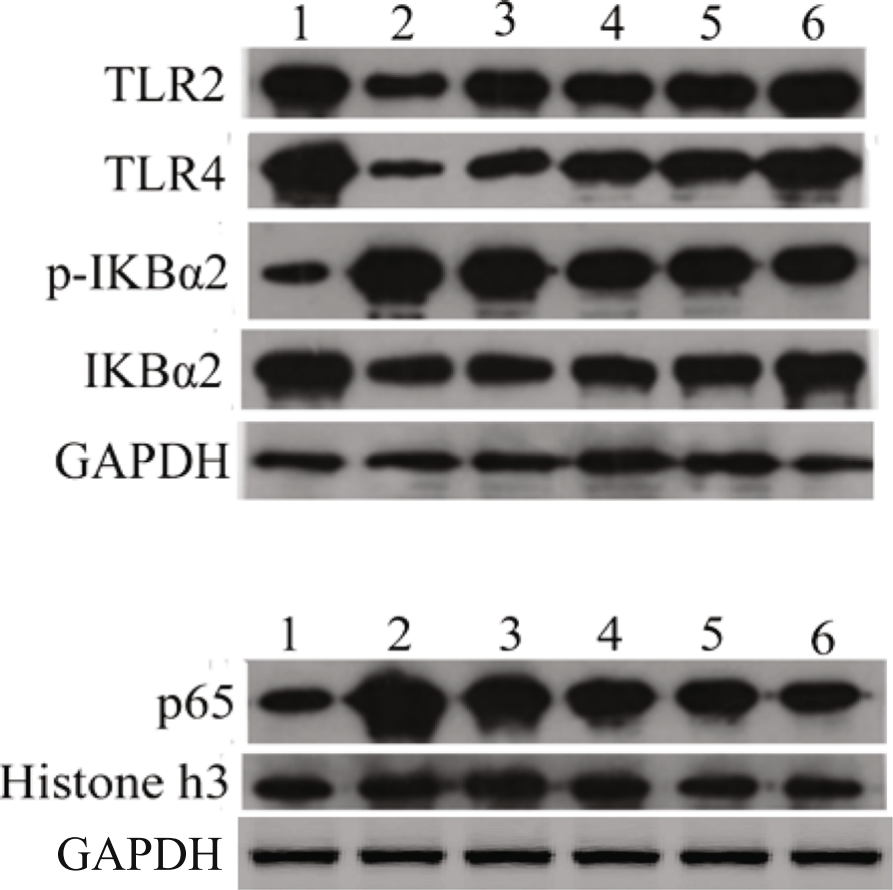
Expression of TLR2, TLR4, p-IkappaB, IkappaB and P65 in lung tissues of COPD rats in each group. 1: Normal control rats, 2: COPD model rats without any treatment, 3: low dose of Feikang granules groups, 4: medium dose of Feikang granules groups, 5: high dose of Feikang granules groups, 6: dexamethasone group

To further elucidate the roles of the TLR4 signaling pathway in the cellular response to LPS in COPD lung tissue, we analyzed the effects of Feikang Granule on the expression of TLR4, TLR2, p-Iκ B, IκB and NF-κ B p65 in COPD model rats. The expression of cytoplasmic protein TLR4 and IκB decreased significantly in the COPD model rats compared to the normal rats (*P* = 0.003, *P* = 0.007), while the expression of cytoplasmic protein p-IκB and nuclear protein p65 increased significantly (*P* = 0.008, P = 0.008) (Fig. 8). Interestingly, the expression of TLR4 and IκB protein in all groups of Feikang granule rats were significantly increased (*P* < 0.05, *P* < 0.01), the expression of nuclear protein NF-κB p65 was significantly decreased (*P* = 0.009), and the expression of p-IκB protein in the middle dose group of Feikang granule was also significantly decreased (P = 0.008).

## Discussion

The present study demonstrates that Feikang granule herbs ameliorate airway blockades and inflammation of smoking-induced COPD rats via the TLR4/NF-κB signaling pathway. Feikang granules significantly reduce LPS induced inflammatory cytokines, such as TNFα, IL-1β, IL-6, and IL-17, released from lung tissue and alveolar macrophages in a dose-dependent manner. These herbal medicines also prevent TLR4 and IκB downregulation and reverse the p-IκB and NF-κB p65 upregulation of the lung tissue in the COPD rats. Feikang granules are found to protect against pulmonary dysfunction and pathological changes in the COPD rats. Therefore, this experimental evidence indicates that Feikang Granules have a solid biological basis for treating COPD patients.

COPD is the most common chronic lung disease in the clinic. The main pathological features of COPD are obstructive bronchiolitis, an increase in mucus secretion, and emphysema. The progression of COPD is the result of an abnormal inflammatory processes of the lung to noxious irritants, predominantly due to cigarette smoking, air pollutants, and biomass fuel. Animal studies of COPD have been developed by concomitant use of different inducers such as cigarette-smoke (CS), lipopolysaccharide (LPS) and porcine pancreatic elastase (PPE), which mimic different aspects of inflammatory responses [19]. It has been reported that a combination of CS and LPS is more time-saving and of higher success rate to produce the clinical characteristics of COPD than each alone [16]. Therefore, the COPD rat model was established by continuous smoking (20 weeks) combined with the tracheal drip of LPS (2 times) in the present study.

As the primary pathogens for COPD, any irritants (CS or LPS) could trigger the body’s natural defense inflammatory mechanism to remove these harmful stimuli. COPD is associated with a switch from a self-limiting inflammatory response to a chronic persistent inflammatory response and involving a variety of inflammatory cells and inflammatory mediators [21]. In bronchoalveolar lavage (BAL) of long-term smokers, it has been reported that an increase of TNF-α, IL-1β, IL-6, IL-8, IL-12 (p40), IL-17, monocyte chemoattractant protein-1 (MCP-1) and macrophage inflammatory protein (MIP)-1α [22]. Although we did not evaluate all inflammatory cytokines/mediators, the selected cytokines, IL-1β, TNFα, IL-6, and IL-17 were found to have significantly increased in our COPD model rats both in secretions and in lung tissue and alveolar macrophages. The Chinese herbs formulae Feikang granules could prevent these cytokine increases in a dose-dependent manner. Since IL-1β and TNFα are pro-inflammatory and are associated with macrophage activation and neutrophilic inflammation, their levels are increased in severe asthma and COPD. It has been proposed that the anti-IL-1 antibody (canakinumab) or anti-TNF antibody (infliximab) could serve(?) as potential treatments for systemic inflammation in asthma or COPD patients [4]. Feikang granules could prevent all cytokine increases and have significant advantages because they are not targeting individual pro-inflammatory cytokines.

The inflammatory mechanisms first recognize invading pathogenic molecular patterns via a group of pattern recognition receptors (PRRs), such as Toll-Like receptors (TLRs). TLRs recognize molecular patterns shared by pathogens and activate inflammatory cells like NF-κB and produce inflammatory cytokines and chemokines to start the resolution process [23]. In the present study, when the LPS is inhaled into the pulmonary alveoli, it is recognized by the cell surface TLRs, thus initiating the immune response against pathogens in macrophages, subsequently producing inflammatory mediators in response to the pathogens [24]. The present study supports that TLRs (TLR4, TLR2) decrease and phosphorylated (active) IκB (p-IκB) and NF-κB p65 increase in COPD model rats, and further increase pro-inflammatory cytokines, while Feikang granules protect these inflammatory responses. However, whether the Feikang granules also trigger the upstream of anti-inflammatory signaling pathways, such as TGF-β, need to be further investigated [18, 25].

Traditional Chinese herbal medicines have been used for thousands of years, most taken orally, to improve the human body’s ability to fight against diseases by improving the internal microenvironment. The herbal medicines with minimal adverse effects could sever as new therapeutic treatment strategies [26]. Actually, some Chinese herbal medicines have been reported to improve pulmonary function and overall quality of life [11, 27–29], and most of our recipes can be found in previous literature. Feikang granules not only prevent the inflammatory responses as the dexamethasone does, but also improve pulmonary function in ways that the dexamethasone cannot. These results suggest that Chinese herbal medicine may provide an alternative for the treatment of COPD. However, further studies are necessary to further investigate the other aspects of Feikang granules, including their possible adverse effects or toxicity in normal or COPD rats.

## Conclusion

In conclusion, present study using cigarette-smoking combined with the LPS rat model demonstrated that the inflammatory responses increased as TNF-α, IL-1β, IL-6, and IL-17 levels elevated and led to the pulmonary dysfunction and pathological changes. The TLR4/NF-kB signaling pathway mediated the mechanism of action. Chinese herbal medicine formula Feikang granules prevented pulmonary inflammation and improved pulmonary function, suggesting that Feikang granules might be an effective treatment for chronic pulmonary diseases, such as COPD.

## Data Availability

The more detailed data used to support the findings of this study are available from the corresponding author upon request.

## Abbreviations

AP1: Activator protein 1
BAL: Broncho-alveolar lavage
BALF: Broncho-alveolar lavage fluid
COPD: Chronic obstructive pulmonary disease
IRF’s: Interferon regulatory factors;
LPS: Lipopolysaccharide
LTA: Lipotoeic acid
NF-κB: Nuclear factor kappa-light-chain-enhancer
MCP-1: Monocyte chemoattractant protein-1
MIP-1α: Macrophage inflammatory protein-1α
PEF: Peak of expiratory flow
PIF: Peak of inspiratory flow
PRRs: Pattern recognition receptors
STAT: Signal Transducers and Activators of Transcription family of transcription factors
TLRs: Toll-like receptors
